# Diet quality and telomere length in older Australian men and women

**DOI:** 10.1007/s00394-016-1326-6

**Published:** 2016-10-26

**Authors:** Catherine M. Milte, Aaron P. Russell, Kylie Ball, David Crawford, Jo Salmon, Sarah A. McNaughton

**Affiliations:** Institute for Physical Activity and Nutrition (IPAN), School of Exercise and Nutrition Sciences, Deakin University, Geelong, Australia

**Keywords:** Diet, Ageing, Telomere length, Diet quality, Mediterranean diet

## Abstract

**Purpose:**

Telomere length is a biomarker of cellular ageing, with longer telomeres associated with longevity and reduced risk of chronic disease in older age. Consumption of a healthy diet may contribute to longevity via its impact on cellular ageing, but studies on diet and telomere length to date have been limited and their findings equivocal. The aim of this study was to examine associations between three indices of diet quality and telomere length in older men and women.

**Methods:**

Adults aged 57–68 years participating in the Wellbeing, Eating and Exercise for a Long Life (WELL) study in Victoria, Australia (*n* = 679), completed a postal survey including an 111-item food frequency questionnaire in 2012. Diet quality was assessed via three indices: the Dietary Guideline Index, the Recommended Food Score, and the Mediterranean Diet Score. Relative telomere length was measured by quantitative polymerase chain reaction. Associations between diet quality and telomere length were assessed using linear regression adjusted for covariates.

**Results:**

After adjustment for age, sex, education, smoking, physical activity, and body mass index (BMI), there were no significant associations between diet quality and relative telomere length.

**Conclusions:**

In a sample of older adults residing in Victoria, Australia, men and women aged 57–68 years with better-quality diets did not have longer telomeres. Further investigation in longitudinal studies will determine whether diet can influence telomere length over time in an ageing population.

**Electronic supplementary material:**

The online version of this article (doi:10.1007/s00394-016-1326-6) contains supplementary material, which is available to authorized users.

## Introduction

The world’s ageing population continues to increase with the number of persons aged 60 years and over expected to exceed the number of children in the world by 2045 [1]. Increased longevity is supporting marked growth in the proportion of adults aged over 85 years [2]. As chronic disease burden increases with age, it is important that health and function are maintained to complement increased longevity. Diet plays a key role in maintaining health in adulthood and may impact on markers of cellular ageing such as telomere length.

Telomeres are repetitive DNA sequences at the ends of eukaryotic chromosomes that undergo attrition each time a somatic cell divides [3]. They prevent loss of genomic DNA at ends of linear chromosomes and protect their physical integrity with cell division and replication, thereby protecting against cell death. Telomere length decreases with age and is considered a biomarker of accelerated cellular ageing. Shorter telomere length is associated with decreased life expectancy and increased risk of chronic disease [4] and was recently described as one of the nine “hallmarks of ageing” [5]. As telomere attrition rate varies between individuals and is increased by inflammation and oxidative stress [4], it is a proposed modifiable lifestyle risk factor for health and longevity in older age. Although there is some genetic regulation of variability of telomere length, it may also be partially explained by lifestyle behaviours, including dietary intake [6]. The intake of various individual nutrients including folate, vitamins C, E, D, A, and β-carotene, magnesium and omega-3 polyunsaturated fatty acids is associated with longer telomere length [6, 7]. However, there has been little investigation into the association between whole diet, or dietary patterns and telomere length.

Whilst previous research into nutrition and health in older age has focussed on the role of individual nutrients or foods, there is increasing interest in dietary pattern analysis as a chronic disease determinant [8]. Dietary patterns can be defined by two approaches: multivariate statistical techniques such as factor or cluster analysis (data-driven approaches), and dietary scoring methods informed by a priori guidelines and recommendations, or diet quality indices. Diet quality indices can assess adherence to dietary guidelines [9], or a particular type of diet such as the Mediterranean diet [10]. Diet quality assessed by adherence to dietary guidelines has been associated with cardiometabolic risk factors [11], and physical and mental health [12, 13], whilst diet quality assessed by adherence to a Mediterranean diet and higher dietary variety has been associated with reduced risk of mortality [14] in older people.

Despite the wide variety of diet quality indices available, few studies have investigated dietary patterns and telomere length in older people [15, 16], and only one has included multiple indices of diet quality [17]. Considering the limited research to date, assessing multiple indices of diet quality may give greater insights into which components or aspects of diet quality have greatest influence on telomere length. The aim of this study was to examine associations between three measures of diet quality and relative telomere length in men and women aged 57 years and over.

## Methods

### Design

This study is based on data from the Wellbeing, Eating and Exercise for a Long Life (WELL) study. The WELL study is a prospective, population-based longitudinal cohort study of nutrition and physical activity behaviours, obesity and quality of life, and the intrapersonal, social and environmental influences on these among mid-aged and older adults [18]. Participants aged between 55 and 65 years, living independently in the community and able to complete a written questionnaire in English, were included. In 2010, participants aged between 55 and 65 years, living in the community in urban or rural Victoria, Australia, were selected from the Australian Electoral Roll, stratified by socioeconomic position using the Socioeconomic Index for Areas score (SEIFA) [19]. Potential participants living in a suburb with a population of less than 1000 overall or <200 in the 55–65 years age bracket were excluded. All eligible suburbs were classified by SEIFA and divided into tertiles (representing low, medium, and high SEIFA). Fourteen postcodes from each SEIFA tertile were randomly selected. From each postcode, 134 participants (equal numbers of men and women) were selected for invitation into the study. A total of 11,256 surveys were distributed to potential participants at baseline in 2010. Of these, 380 were returned as undeliverable and 95 were returned from individuals outside the age bracket. In total, 4082 completed surveys were returned at baseline (response rate 38%). Participation was voluntary, and informed consent was obtained by return of the survey. In 2012, participants who agreed to take part in a follow-up were sent a similar survey (*n* = 3368). Of these, 2758 completed surveys were returned (response rate 82%). Data were collected at the same time of year in 2010 and 2012 to negate any potential seasonal effects. Ethical approval for the study was obtained from the Deakin University Human Research Ethics Committee (2009-105). Full details of the study have been described elsewhere [18].

In 2012, fasting blood samples were collected in a subgroup of volunteers as part of the WELL Heart Health sub-study. Volunteers invited to take part in this subgroup needed to be dwelling in suburbs classified as urban or urban fringe at the time of the 2012 survey (to allow access to pathology centres where blood samples were taken). A sample of *n* = 1283 (667 women and 616 men) were invited to participate via an invitation pack sent out to their postal address, with 884 consenting to take part (69% response rate) and 777 providing a blood sample. A total of 768 whole blood samples were collected in EDTA-coated tubes, and telomere length was successfully measured in 764 samples.

### Telomere length

Relative telomere length was measured in whole blood by quantitative polymerase chain reaction (qPCR) [20]. All assays were processed by the same technician under identical conditions. DNA was extracted using the QIAamp DNA blood mini kit (cat # 51106; Qiagen, Clifton Hill, VIC) according to the manufacturer’s protocol.

Relative telomere length was determined using qPCR to compare telomere repeat sequence copy number (*T*) to a single-copy gene copy number (*S*) (*T*/*S*), as previously described [20, 21]. The primers for the telomere qPCR were tel-c 5′-CGGTTTGTTTGGGTTTGGGTTTGGGTTTGGGTTTGGGTT-3′, and tel-g 5′-GGCTTGCCTTACCCTTACCCTTACCCTTACCCTTACCCT-3′, and were used at final concentrations of 100 and 900 nM, respectively. The primers for the single-copy gene, human beta-globulin, were h-βgFor 5′-GCTTCTGACACAACTGTGTTCACTAGC-3′ and h-βgRev 5′-CAC CAA CTT CAT CCA CGT TCA CC-3′, both used at 300 nM. Each 20-µl qPCR reaction contained 1 U AmpliTaq Gold 360 DNA polymerase with 1 × AmpliTaq Gold 360 buffer (cat # 4398892; Life Technologies), 2 mM MgCl, 1 mM dithiothreitol (cat # 43186; Sigma-Aldrich, St Louis, MO, USA), 1 M betaine (cat # B0300-5VL; Sigma-Aldrich), 0.2 mM dNTP mix (cat # 18427-088 l; Life Technologies), 0.01 mM SYTO 9 Green Fluorescent Nucleic Acid Stain (cat # S34854; Life Technologies), 5 ng DNA, and the primers of interest. A seven-point standard curve was generated using serial dilutions from 40 to 0.625 ng of DNA from a pooled sample. Aliquots were prepared for each of the serial dilutions so that the same samples were used for standard curve on each PCR plate without freeze thawing. The standard curves for telomere and beta-globulin were both included on each PCR plate. Each standard and sample was measured in duplicate in 384-well plates (cat # HSP-3801, Bio-Rad, Hercules, CA, USA) using the Bio-Rad C1000 thermal cycler. Cycling conditions consisted of one cycle at 95 °C for 10 min, followed by 40 cycles at 95 °C for 5 s, 60 °C for 20 s, and 72 °C for 60 s. A dissociation curve was generated to show that only one product had been amplified during the PCR.

To determine the relative telomere length, the telomere and human beta-globulin standard curves were plotted as log[DNA] concentration against the PCR Ct value. A linear straight line (*y* = *mx* + *C*) was generated for the telomere and beta-globulin standard curves, and the log[DNA] concentration for telomere and beta-globulin was determined for each unknown sample. Relative telomere length was expressed as telomere-to-standard single-copy gene ratio (*T*/*S* ratio). The inter-assay coefficient of variation was 3.1% whilst the intra-assay coefficient of variation was 0.8%.

### Dietary intake

Usual dietary intake at baseline was assessed using an 111-item food frequency questionnaire (FFQ) [22, 23], which assessed self-reported intake of food and beverages over the last 6 months. The FFQ has been previously used in other national studies [24–26]. We have also previously used this questionnaire to assess dietary patterns and diet quality and have demonstrated it is a good predictor of health outcomes [9]. The survey included seven additional validated short questions on food habits including salt use (during and after cooking), type of milk and bread consumed, trimming the fat from meat and daily fruit and vegetable consumption [26, 27]. Frequencies were converted into daily equivalents for analysis [28].

### Diet quality

Diet quality was assessed using three previously developed indices: the Dietary Guideline Index (DGI), the Recommended Food Score (RFS), and the Mediterranean Diet Score (MDS). The indices were adapted for use with the data from the FFQ. The DGI is an updated version of a previous index developed to reflect Australian guidelines for optimal eating patterns which was shown to be a good measure of diet quality [9]. The index was updated to reflect the 2013 Australian Dietary Guidelines [29]. For each dietary guideline component, indicators from the FFQ were identified and food groupings determined. Age- and sex-specific scoring cut-offs for the five core food groups (vegetables, fruits, grains, meat and alternatives, and dairy), fluids, and discretionary foods were devised. Discretionary foods (also commonly known as “extra” foods) are foods that are not essential to provide nutrient requirements due to the high content of sugar, fat, and salt such as soft drinks, cordials, fruit juice drinks, chips, confectionary, chocolate, hamburgers, meat pies, pizza, cakes and muffins, pies and pastries, biscuits, and alcoholic beverages [29]. Items referring to whole-grain cereals, lean protein, reduced/low-fat dairy, unsaturated fats, and dietary variety were included in the index. A total of 13 components were included in the updated DGI. Each component of the DGI was scored proportionally from 0 to 10, where 10 indicated that a participant was fully meeting the recommendation. The total score was the sum of 13 items so that the diet score had a possible range of 0–130, with higher scores reflecting greater compliance with the Australian Dietary Guidelines. The previous version of the DGI was evaluated in the Australian population and shown to be related in predicted directions to sociodemographic factors, health behaviours, self-assessed health, and intakes of key nutrients [9, 13].

The RFS is a food-based score which assesses the frequency of consumption of a range of foods considered to be consistent with existing dietary guidelines [30]. The RFS has been previously validated and is associated with biomarkers of dietary intake, chronic disease, and mortality [30, 31]. Participants are allocated a score of 1 for each recommended food consumed more than once per week. A total of 49 foods listed in the FFQ were considered as recommended (consistent with dietary guidelines: fruit, vegetable, whole grain, lean meat and alternatives, and low-fat dairy groups) resulting in scores ranging from 0 to 49, with higher scores associated with a wider variety of consumption of recommended foods and greater diet quality.

Adherence to a Mediterranean diet was assessed using the most commonly used MDS developed by Trichopoulou et al. [10]. This index has demonstrated validity through reported relationships with nutrient biomarkers [32] and health outcomes [14, 33–35]. The MDS includes vegetables (excluding potatoes), legumes, fruits and nuts, cereals, fish and seafood, dairy products, meat and meat products, and alcohol. In accordance with standard scoring techniques, we calculated sex-specific medians for frequency of intake of these items. For vegetables, legumes, fruits and nuts, cereals, fish and seafood, participants with an intake above the median were assigned a score of 1. For dairy products and meat and meat products, participants with an intake below the median were assigned a score of 1. For alcohol, a score of 1 was assigned for low to moderate intake (intake of no more than 2 times/day) and a score of 0 for no alcohol intake or intake >2 times per day. Supplementary Table S1 (Online Resource 1) shows the median frequency of intake for the eight food items included in the MDS. Total score was calculated as the sum of all components, and ranged from 0 to 8, with higher scores reflecting greater adherence to a Mediterranean diet. An olive oil consumption or monounsaturated-to-saturated fat ratio item is also usually included. We were unable to include this item as it was not assessed in the FFQ. This adapted version of the MDS was previously shown to be associated with health-related quality of life in this cohort [13].

### Covariates

Participant characteristics were collected during the postal survey, including age, country of birth, marital status, retirement status, and smoking status. Menopausal status was collected in women. Highest education level achieved was collected as a measure of socioeconomic position. Self-reported height and weight were collected for calculation of body mass index (BMI; kg/m^2^). Total physical activity was captured using the self-administered International Physical Activity Questionnaire (IPAQ-L), which has demonstrated validity and reliability in a 12-country, 14-site study [36]. The IPAQ-L assesses duration, frequency, and intensity of leisure, work, commuting, and household/yard activities during the past 7 days. Responses were converted into total metabolic equivalent of task (MET) hours per week with moderate physical activity at 3 MET and vigorous physical activity at 6 MET.

### Statistical analysis

Participants missing >10% responses on the FFQ, 1 or more responses to the dietary habits questions, or the *T*/*S* ratio or covariates were excluded from analysis. Differences between included and excluded participants were explored using *t* tests for normally distributed variables, the Wilcoxon rank sum test (Mann–Whitney test) for non-normally distributed variables, and Chi-square tests for categorical variables. Summary of participant characteristics was presented by quartile of relative telomere length. For the main analysis, associations between diet quality and relative telomere length were assessed via linear regression with relative telomere length as a continuous variable. Covariates adjusted for in regression models were selected based on previous literature [9, 37–39], and only those associated with the outcome or exposure were included in the models. Multivariable linear regression models were adjusted for age, sex, and education (model 1), with further adjustments made for smoking, total physical activity (model 2) and BMI (model 3). BMI was included in the final adjustment as obesity may attenuate associations between diet quality and health outcomes, and so the results of analyses with (model 3) and without BMI (model 2) could be assessed separately. StataSE version 13.1 (StataCorp, TX, USA) was used for all statistical analyses. *P* < 0.05 was considered significant.

## Results

There was complete data available for analysis on 679 participants (Table 1). Characteristics of participants included in the analysis and participants with incomplete data not included in the analysis are summarised in supplementary Table S2 (Online Resource 2). The age of participants included in analysis ranged from 57 to 68 years. Women had a longer relative telomere length than men (mean ± SD; 1.01 ± 0.14 vs. 0.97 ± 0.14, *P* < 0.01, respectively). Relative telomere length did not differ by age, smoking status, BMI, or physical activity.

**Table 1:** Characteristics of 679 men and women from the WELL Study by quartile of relative telomere length, Victoria, Australia, 2012

	Total	*Q*1	*Q*2	*Q*3	*Q*4
*T*/*S* ratio, mean (SD)	0.99 (0.14)	0.82 (0.08)	0.94 (0.03)	1.04 (0.03)	1.17 (0.07)
*T*/*S* ratio, range	0.43–1.4	0.43–0.89	0.89–0.99	0.99–1.09	1.09–1.4
DGI-2013, mean (SD)	88.3 (13.0)	86.6 (12.9)	88.0 (13.3)	88.6 (12.4)	90.1 (13.2)
RFS, mean (SD)	24.7 (7.91)	24.5 (8.15)	24.9 (7.50)	24.4 (8.02)	25.2 (8.00)
MDS, mean (SD)	4.25 (1.56)	4.28 (1.52)	4.34 (1.64)	4.04 (1.60)	4.34 (1.45)

	Mean (SD)	Mean (SD)	Mean (SD)	Mean (SD)	Mean (SD)	*P* for trend
Age	62.7 (3.07)	63.0 (3.14)	62.9 (2.97)	62.4 (3.03)	62.6 (3.14)	0.507
BMI	26.7 (4.94)	27.4 (5.49)	25.9 (4.30)	26.8 (4.65)	26.8 (5.16)	0.116
Total physical activity^a^	97.2 (84.2)	99.5 (86.6)	97.8 (79.9)	105 (96.7)	86.4 (71.1)	0.204

	*n* (%)	*n* (%)	*n* (%)	*n* (%)	*n* (%)	
Sex, men	330 (49)	95 (56)	81 (48)	82 (48)	64 (38)	0.011
Smoking						0.542
Never smoked	396 (58.3)	95 (55.1)	92 (54.1)	105 (61.8)	104 (61.5)	
Former smoker	228 (33.6)	57 (33.5)	64 (37.7)	55 (32.4)	52 (30.8)	
Daily smoker	55 (8.10)	18 (10.6)	14 (8.24)	10 (5.88)	13 (7.69)	
Country of birth^b^						0.559
Australia	515 (80.0)	131 (77.1)	133 (78.2)	131 (77.1)	120 (71.4)	
UK	54 (7.96)	16 (9.41)	10 (5.88)	14 (8.24)	14 (8.33)	
Other	109 (16.1)	23 (13.5)	27 (15.9)	25 (14.7)	34 (20.2)	
Marital status^b^						0.604
Married/defacto	520 (76.8)	131 (77.1)	124 (73.8)	136 (80.0)	129 (76.3)	
Separated/divorced	79 (11.7)	22 (12.9)	22 (13.1)	18 (10.6)	17 (10.1)	
Widowed	38 (5.61)	8 (4.71)	11 (6.54)	9 (5.29)	10 (5.92)	
Never married	40 (5.90)	9 (5.29)	11 (6.54)	7 (4.12)	13 (7.69)	
Education						0.011
Up to 10 years	165 (24.3)	45 (26.5)	40 (23.5)	43 (25.3)	37 (21.9)	
12 years/trade/certificate	232 (34.2)	66 (38.8)	68 (40.0)	56 (32.9)	42 (24.8)	
University degree	282 (41.5)	59 (34.7)	62 (36.5)	71 (41.8)	90 (53.3)	

*BMI* body mass index, *DGI* Dietary Guideline Index, *MDS* Mediterranean Diet Score, *RFS* Recommended Food Score, *T/S ratio* relative telomere length, *WELL* Wellbeing, Eating and Exercise for a Long Life

^a^Reported as MET hours per week

^b^Reduced sample size for variable due to missing data

Table 2 shows the association between diet quality assessed by the DGI-2013, RFS and MDS, and relative telomere length. There were no associations between diet quality and relative telomere length.

**Table 2:** Multivariable-adjusted regression coefficients and 95% CI per unit increase in diet quality score for relative telomere length (*T*/*S* ratio) (*n* = 679)

Telomere length	DGI (*n* = 679)		MDS (*n* = 679)		RFS (*n* = 679)	
	*β* (95% CI)	*P* value	*β* (95% CI)	*P* value	*β* (95% CI)	*P* value
Model 1	0.001 (−0.000, 0.001)	0.17	−0.001 (−0.008, 0.006)	0.77	−0.0004 (−0.002, 0.001)	0.60
Model 2	0.001 (−0.000, 0.001)	0.17	−0.001 (−0.008, 0.006)	0.79	−0.0004 (−0.002, 0.001)	0.61
Model 3	0.001 (−0.000, 0.001)	0.17	−0.001 (−0.008, 0.006)	0.82	−0.0004 (−0.002, 0.001)	0.62

*DGI* Dietary Guideline Index, *MDS* Mediterranean Diet Score, *RFS* Recommended Food Score, *BMI* body mass index

Model 1: controlling for age, sex, and education, Model 2: model 1 + smoking and total physical activity, Model 3: model 2 + BMI

## Discussion

In this study of 679 community-dwelling older men and women from the WELL study, greater diet quality assessed via the DGI-2013, RFS, and MDS was not cross-sectionally associated with longer relative telomere length. Previous studies have reported cross-sectional associations between higher intakes of vegetables and lower intakes of saturated fat and sugar-sweetened soda and longer telomere length [40, 41]. Reported associations between diet quality and telomere length have been limited, with only one study investigating associations between multiple dietary indices and telomere length. In 4676 women, aged 42–70 years from the Nurses’ Health Study, higher scores on the Mediterranean diet and Alternate Healthy Eating Index were associated with longer telomere length. However, no associations between prudent and Western dietary patterns and telomere length were observed [17]. A cross-sectional study of 217 men and women aged 71–87 years reported that a greater adherence to a Mediterranean diet was associated with longer leucocyte telomere length and higher telomerase activity, a relationship moderated by inflammation [15]. Following a 5-year Mediterranean diet intervention, an association between TL and changes in adiposity indices was observed in 521 men and women aged 55–80 years [42]. Furthermore, in the same cohort, telomere shortening appeared to be attenuated with greater adherence to the Mediterranean diet in people carrying the Pro12Ala polymorphism in the peroxisome proliferator-activated receptor gamma 2 (*PPARy*2) gene [43].

Studies investigating dietary patterns and telomere length longitudinally have been limited to date. A randomised crossover intervention of 20 older adults found reduced telomere shortening following a Mediterranean diet for 4 weeks compared to a saturated fatty acid diet and low-fat high-carbohydrate diet [44]. More recently, a randomised intervention trial of the Mediterranean diet in 520 participants aged 55–80 years reported greater adherence to a Mediterranean diet was associated longer telomeres in women only at baseline [45]. However, no beneficial effects for the Mediterranean diet on telomere length were observed at 5-year follow-up, and a higher risk of telomere shortening was observed in the Mediterranean diet and nuts group.

Associations between inflammatory dietary patterns derived by principal component analysis and leucocyte telomere length in 840 men and women aged 45–84 years have been investigated [16]. No relationship between telomere length and a “fats and processed meat” pattern or a “whole grains and fruit” pattern was observed, despite these patterns being associated with C-reactive protein, interleukin-6, and homocysteine [16]. However, an association between intake of processed meat and shorter telomeres was reported. A recent Mediterranean diet intervention study of 520 participants at high cardiovascular risk reported a pro-inflammatory diet characterised by a dietary inflammatory index was associated with shorter telomeres cross-sectionally and after a 5-year follow-up [46].

Previous studies have also investigated associations between healthy lifestyle and telomere length. In 612 patients with advanced prostate cancer and 1049 matched controls, the sum score of a healthier lifestyle and diet factors (low or no cigarette use, higher fruit and vegetable intake, lower BMI, more physical activity), correlated with longer telomere length; no correlations between telomere length were observed when these factors were analysed individually [47]. In patients with low-risk prostate cancer, a comprehensive lifestyle intervention (changes to diet, physical activity, stress management, and social support) resulted in increased telomere length and telomerase activity, when comparing baseline and 5-year follow-up values. Adherence to the lifestyle changes was also significantly associated with relative telomere length when adjusted for age and length of follow-up [48]. Similarly, in a cohort of 5862 women (aged mean 58.7 ± 0.09 years), increased telomere length was associated with adherence to a healthy lifestyle; the later defined using five components including smoking, physical activity, adiposity, alcohol use, and diet [49].

Previous studies have reported inverse correlations between age and telomere length [15–17, 50, 51]. However, no association was observed between age and telomere length in the current study. The age range of our sample of 57–68 years was considerably narrower than that of previous studies, with reported age ranges of 42–70 [17], 71–87 [15], 45–84 [16], 1–96 [50], and 18–90 [51] years. In the present study, the *T*/*S* values ranged between 0.43 and 1.40 which are similar to values observed previously within the same age range [50]. Our lack of observation of an association between age and telomere length is most likely due to the small age range. This is not without precedence and there appears to be limited correlation between age and telomere length when analysed within narrow age ranges of 40–44 and 60–64 years [52].

Diet quality has been shown to be associated with mortality [14] and reduced risk of chronic diseases such as cardiovascular disease and diabetes [53], all outcomes that have been associated with shortening of telomeres [4]. Previously, it has been proposed that consumption of a healthier diet would protect against age-related telomere loss through reduced oxidative stress and inflammation [6]. The DGI was associated with lower intakes of energy, total fat and saturated fat, and higher intakes of fibre, β-carotene, vitamin C, folate, calcium, and iron previously [9]. The RFS was associated with intake of similar nutrients and antioxidants and also reduced markers of chronic disease and inflammation, including serum homocysteine, C-reactive protein, plasma glucose, total serum cholesterol, and blood pressure in previous research [31]. The Mediterranean diet is also proposed to reduce disease risk through its anti-inflammatory and anti-oxidative properties [54]. In summary, although previous research demonstrates the potential for all three indices used in the current study to assess long-term adherence to diets associated with reduced inflammation and oxidative stress, no significant associations with telomere length were observed.

These findings are generalisable to community-dwelling men and women around the “peri-retirement” stage. However, it should be noted that WELL study participants reported higher scores on the RAND 36-item survey [13] compared to other Australian population-based samples [55, 56], and may therefore represent a group with better health status than the Australian population. The samples are also not reflective of the oldest old or specific clinical populations, and therefore, these findings may not be applicable across the older age range in a broader sense. However, there are challenges to assessing telomere length in older age, as the upper age limit for reliable assessment of telomere length is 75 years [57]. Telomere length instability and “survivor bias”, where people who live longer are suspected to possess longer telomeres and some resilience to chronic disease, may contribute to a lack of association between telomere length and mortality observed above this age [57, 58]. However, this is unlikely to have played a role in the lack of associations observed in the current study.

The strengths of our study include the well-characterised sample, detailed assessment of dietary intake and potential confounders and the use of three validated diet quality indices. However, the study has some limitations that should be considered. Only a subsample of the original cohort provided a blood sample, which may have created a selection bias. Dietary intake was assessed by a non-quantified FFQ, which did not allow for adjustment of energy intake in statistical analysis. However, key determinants of energy intake were taken into account by adjusting for sex, BMI, and physical activity. Although a range of potential covariates were tested for inclusion in the regression models, it is possible some residual confounding remained. Finally, the relatively modest sample size may have limited our ability to detect the small associations reported by previous studies, although this is unlikely since some of those studies were conducted in a sample of similar size to the current study [15, 17].

A potential limitation of studies investigating the Mediterranean diet in non-Mediterranean samples is the lack of foods and eating behaviours truly reflective of a traditional Mediterranean diet. However, studies in Australian [59, 60] and UK samples [61] have reported the presence of a Mediterranean-style pattern. Our choice of Mediterranean Diet Index included sample-specific median intakes of vegetables, legumes, fruits and nuts, cereals, and fish as cut points for scoring. Although this enables adaptation of the score for a non-Mediterranean population, it likely reflects a dietary pattern with substantially lower absolute intakes of these foods compared to typical of Mediterranean populations as supported by a recent Australian study [62]. An olive oil or monounsaturated fat component was not included in the MDS in this study due to limitations in the FFQ. However, some controversy exists regarding the inclusion of this item in Mediterranean diet assessment in non-Mediterranean populations, as olive oil consumption is typically low and monounsaturated-to-saturated fat ratio instead reflects animal fat intake, which is low in the typical Mediterranean diet [63].

Although telomere length is thought to vary depending on exposure to environmental factors, telomere length is also determined by genetics [64] and shows inter-individual variation from birth [65]. Thus, attempts to determine the association between diet and telomere attrition should ideally measure within-person change in telomere length over long periods of time. The current study was cross-sectional and was therefore unable to assess within-person change in telomere length. This may have limited the ability to detect significant associations in the current study. It should be noted that all previous studies investigating dietary patterns and telomere length have also been cross-sectional. Therefore, inconsistencies in the evidence to date including the current study may be related to the fact that telomere length shows inter-individual variation and telomere length at any age relates to a combination of telomere length at birth and subsequent rate of change [66]. Considering the variation in findings of this and previous studies, it is critical that future studies are longitudinal in study design and include assessment of telomere length over time to determine a true measure of biological ageing.

### Conclusion

In a sample of adults residing in Victoria, Australia, men and women aged 57–68 years with better-quality diets did not have longer telomeres. Further investigation of telomere length over time in longitudinal studies will determine whether dietary patterns are associated with cellular ageing in an older population.

## Electronic supplementary material

Below is the link to the electronic supplementary material.

Supplementary material 1 (PDF 93 kb)

Supplementary material 2 (PDF 93 kb)
