# Supplementary material for: Diet quality and telomere length in older Australian men and women
Source: Eur J Nutr. 2016 Oct 26;57(1):363–72. doi: 10.1007/s00394-016-1326-6 (PMC5847066; doi:10.1007/s00394-016-1326-6)
Supplement: Supplementary file 1 — Supplementary material 1 (PDF 93 kb) [file 394_2016_1326_MOESM1_ESM.pdf]

Title: Diet quality and telomere length in older Australian men and women

Journal title: European Journal of Nutrition

Catherine M Milte, Aaron P Russell, Kylie Ball, David Crawford, Jo Salmon and Sarah A McNaughton

Centre for Physical Activity and Nutrition Research, School of Exercise and Nutrition Sciences, Deakin University, Geelong, Victoria, Australia

Email: catherine.milte@deakin.edu.au

S1. Median and mean (SD) daily equivalent intake frequencies of food groups from the MDS of 679 Men and Women from the WELL Study by quartile of relative telomere length, Victoria, Australia, 2012.

	Total		Q1		Q2		Q3		Q4	
	Median	Mean (SD)	Median	Mean (SD)	Median	Mean (SD)	Median	Mean (SD)	Median	Mean (SD)
Vegetables	5.55	5.97 (3.15)	5.16	5.93 (3.81)	5.60	5.90 (2.70)	5.39	5.49 (2.23)	5.99	6.54 (3.55)
Legumes	0.07	0.17 (0.34)	0.67	0.21 (0.52)	0.67	0.16 (0.21)	0.67	0.15 (0.27)	0.67	0.17 (0.27)
Fruit and nuts	3.28	3.72 (2.57)	3.34	3.75 (2.56)	3.27	3.72 (2.61)	2.92	3.51 (2.48)	3.49	3.90 (2.64)
Cereals	1.92	2.11 (1.16)	2.07	2.13 (1.11)	1.86	2.09 (1.18)	2.14	2.22 (1.11)	1.85	1.99 (1.24)
Fish	0.28	0.38 (0.35)	0.29	0.41 (0.38)	0.28	0.37 (0.29)	0.21	0.36 (0.34)	0.28	0.39 (0.36)
Dairy	1.64	1.83 (1.18)	1.71	1.90 (1.16)	1.56	1.71 (1.15)	1.74	1.94 (1.20)	1.63	1.78 (1.19)
Meat	0.78	0.91 (0.67)	0.84	0.99 (0.83)	0.77	0.89 (0.57)	0.84	0.95 (0.67)	0.76	0.82 (0.54)
Alcohol	0.50	0.75 (0.92)	0.56	0.87 (1.10)	0.50	0.69 (0.76)	0.43	0.74 (0.98)	0.50	0.71 (0.79)
MDS	4	4.25 (1.56)	4	4.28 (1.52)	4	4.34 (1.64)	4	4.04 (1.60)	4	4.34 (1.45)

MDS, Mediterranean diet score; WELL, Wellbeing, Eating and Exercise for a Long Life
