# Supplementary material for: Diet quality and telomere length in older Australian men and women
Source: Eur J Nutr. 2016 Oct 26;57(1):363–72. doi: 10.1007/s00394-016-1326-6 (PMC5847066; doi:10.1007/s00394-016-1326-6)
Supplement: Supplementary file 2 — Supplementary material 2 (PDF 93 kb) [file 394_2016_1326_MOESM2_ESM.pdf]

Title: Diet quality and telomere length in older Australian men and women

Journal title: European Journal of Nutrition

Catherine M Milte, Aaron P Russell, Kylie Ball, David Crawford, Jo Salmon and Sarah A McNaughton

Centre for Physical Activity and Nutrition Research, School of Exercise and Nutrition Sciences, Deakin University, Geelong, Victoria, Australia

Email: catherine.milte@deakin.edu.au

S2. Characteristics of Men and Women from the WELL Study included and excluded from analysis, Victoria, Australia, 2012.

	Included (n=679)	Excluded (n=3403)	
	Mean (SD)	Mean (SD)	P value
Age	62.7 (3.07)	62.7 (3.13)	0.72
BMI[b]	26.7 (4.94)	27.2 (5.07)	0.01
Total physical activity[ab]	97.2 (84.2)	112.8 (99.9)	>0.001
	n (%)	n (%)	
Sex, men	322 (47.4)	1622 (47.7)	0.91
Smoking[b]			
Never smoked	396 (58.3)	1019 (49.3)	>0.001
Former smoker	228 (33.6)	854 (41.3)	
Daily smoker	55 (8.1)	193 (9.3)	
Country of birth[b]			
Australia	515 (75.9)	1719 (82.6)	0.75
UK	54 (8.0)	142 (6.8)	
Other	109 (16.1)	213 (10.2)	
Marital status			
Married/defacto[b]	520 (76.6)	1612 (77.5)	0.747
Separated/ divorced	79 (11.6)	250 (12.0)	
Widowed	38 (5.6)	104 (5.0)	
Never married	40 (5.9)	103 (5.0)	
Education[b]			
Up to 10 yrs	165 (24.3)	1319 (39.7)	>0.001

12 yrs/ trade/ certificate	232 (34.2)	1204 (36.2)
University degree	282 (41.5)	803 (24.1)

BMI, body mass index; WELL, Wellbeing, Eating and Exercise for a Long Life.

[a]Reported as MET hours per week. [b]Reduced sample size for variables due to missing data.
